# “On Edge All the Time”: Mixed-Status Households Navigating Health Care Post Arizona's Most Stringent Anti-immigrant Law

**DOI:** 10.3389/fpubh.2018.00383

**Published:** 2019-01-15

**Authors:** Sofía Gómez, Anna O. O'Leary

**Affiliations:** ^1^Department of Community, Environment & Policy, Mel and Enid Zuckerman College of Public Health, University of Arizona, Tucson, AZ, United States; ^2^Mexican American Studies, College of Social and Behavioral Sciences, University of Arizona, Tucson, AZ, United States

**Keywords:** immigration policy, mixed-status households, immigrant health, access to care, SB1070, undocumented immigrants, health promotion, qualitative research

## Abstract

Arizona's state-level policies restricting undocumented immigrants' access to public benefits continue to have implications on mixed-status households' accessibility to care. More notably, the effects of prolonged stress, anxiety and trauma remain unaddressed whilst mental health services continue to be absent. This article examines the healthcare experiences of mixed-status households after Arizona's SB1070 (“Support Our Law Enforcement and Safe Neighborhoods Act”) was passed. Arizona Senate Bill 1070 (SB1070) was state legislation empowering police to detain individuals unable to prove their citizenship upon request. Of particular interest is how households navigate accessibility to care when members have varied immigration statuses, hence, varied healthcare availability. Interviews with 43 households in Tucson, Arizona, 81% of which had at least one undocumented member, reveal barriers and promoters to care. Barriers include complexity of applications, fear and trepidation in seeking care. Promoters include discount care programs that are a vital source of care as well as discretionary practices exercised by front-line staff. Findings have implications beyond Arizona as immigrants settle in new destination states while the current Trump administration borrows from Arizona's anti-immigrant policies.

## Introduction

Immigration policy and policies concerning undocumented immigrants are highly contentious issues in the U.S. The 2016 national election emboldened anti-immigrant rhetoric and climate that Arizona epitomized in 2010 with the passage and enactment of Arizona Senate Bill 1070 (SB1070), “Support Our Law Enforcement and Safe Neighborhoods Act” (1, 2). Arizona's SB1070 was proposed to encourage undocumented immigrants to “self deport” by restricting their access to public goods and services (3). Although three out of the four provisions of Arizona's SB1070 law were found unconstitutional and struck down by the U.S. Supreme Court, the remaining provision was among the most controversial known as “show me your papers” clause (4). It requires law enforcement to verify the immigration status of persons suspected to be “undocumented.” Ultimately in 2016, the American Civil Liberties Union (ACLU) and the Arizona Attorney General's office reached an agreement to set guidelines for law enforcement officers to follow when encountering undocumented immigrants (5). Although not a reversal of law, the guidelines set limits to prevent racial profiling and making unlawful stops based on a person's *perceived* immigration status.

National debates gave rise and have reignited anti-immigrant rhetoric. Tougher immigration enforcement and restrictive policies continue under the new administration, giving rise to greater uncertainty, which may drive more mixed-status households into the shadows (6). Moreover, the daily anxiety of being detained and deported creates an environment of fear and emotional distress for immigrant communities (7–10). Immigrants' fear of deportation and social stigmatization impede access to health care and make them more vulnerable to chronic illnesses (11, 12). These factors may contribute to immigrant households forgoing or delaying needed medical services, even if members in these households are eligible for services.

Furthermore, when the primary family wage earner is deported during Immigration Custom Enforcement's (ICE) removal operations, the remaining family members suffer economic hardships (10, 13). Particularly affected are children of mixed-status households where one or both parents are non-citizens, either undocumented or permanent legal resident, and where one or more children are U.S. citizens (14–16). Children born in the U.S. to immigrant parents are entitled to health services and programs, yet are much less likely to access these services because of their parents' immigration status (16–18). A Pew Hispanic Research report indicated that mixed-status households have grown nationally from 2.7 million in 2003 to 4.5 million in 2010 (19). The exact number of mixed-status households in Arizona is unknown.

Title IV of the Personal Responsibility Work Opportunity Reconciliation Act (PROWA) of 1996 restricted federal public benefits to undocumented immigrants (20, 21). With the exception of emergency Medicaid and some public health services (i.e., immunizations, screening and treatment of communicable diseases), undocumented immigrants remain ineligible for public health benefits (22). Under the Affordable Care Act (ACA), undocumented immigrants were also excluded and are unable to purchase individual private coverage through the marketplace (22). State level immigration legislation further restricted access to state and local public services (23–25). Although undocumented immigrants do not qualify for public benefits, immigrant families may be comprised of eligible household members entitled and eligible for such programs. With the expansion of Arizona Health Care Cost Containment System (AHCCCS) in 2014 and the Affordable Care Act we expected to see an increase in enrollments yet Latinos remain the lowest group to enroll in ACA in Arizona (26). These exclusionary public policies lead to structural and institutional barriers that increase health inequity in immigrant communities.

Health services are limited to available programs provided by local municipalities and non-governmental organizations. Federally Qualified Health Centers (FQHCs) play a critical role in the provision of primary care services to medically underserved communities including immigrants (27). These centers are particularly attractive due to their location, value of cultural competence, sliding fee scales for uninsured and their overall mission to serve the poor, uninsured, and vulnerable population. According to a recent report by the Urban Institute Health Policy Center dependence on these safety nets have grown in recent years, making FQHCs particularly under-funded to serve the growing number of Medicaid expansion and ACA recipients (28–30).

This article examines the health care experiences of mixed-status households in Arizona's anti-immigrant political environment. For this study, the “household” is defined as the most fundamental unit of social groupings. A household is identified in part by co-residence of its members, and the tasks that work to sustain the social grouping (31). This study took place 5 years after SB1070, from September 2015 through July 2016, in Tucson Arizona. Of particular interest was how the household unit navigates accessibility to care when members have varied immigration statuses hence varied health care eligibility, affecting their health care availability. Health care accessibility (or access to care) is defined as the presence of a location or person that family members go to for routine preventative care, urgent care, and medical treatment when sick and/or to seek health advice/consult. This article provides an insight of health-seeking behaviors of mixed-status households to better assist outreach efforts to this population.

## Materials and Methods

Semi-structured interviews were used to identify barriers, promoters and strategies used by members of immigrant households to obtain health care. The interview consisted of qualitative and quantitative questions collecting demographic data from participants and their household, immigration status, health care accessibility, and program utilization by the interviewee and household members. The University of Arizona's Institutional Review Board approved this study. Due to the vulnerability and predicament of mixed-status households, special considerations were taken to safeguard confidentiality. Specifically a written signed consent was seen as a risk as it would contain interviewee identifying information (a person's name). An oral disclosure that included all the appropriate elements of consent was deemed appropriate and granted by the University of Arizona's Institutional Review Board. All study participants were consented verbally in lieu of a signed consent form. Additionally it was anticipated that Spanish would be the preferred language therefore all oral and written information was made available in Spanish as well as in English for those who preferred it.

A specific disclosure protocol was followed in obtaining the verbal consent:

- Only the PI was involved in the consent and interview process.
- A step that was taken to minimize the possibility of coercion or undue influence included allowed time for questions from recruited participants about the consent process before proceeding to the interview session.
- Eligible participants were given a disclosure form before proceeding to the interview session via an IRB approved “Disclosure & Waiver of Consent Script.”
- During the interview session eligible participants had an opportunity to ask questions and consider and/or reconsider if they wished to participate; they could decline and/or stop the interview at any time.
- Participants could have opted NOT to have the interview session recorded.
- A verbal “yes” to conduct the interview was documented by PI upon verbal consent by participant.
- Written information describing the research was provided to each participant who so wished to obtain it in writing.

### Questionnaire

The questionnaire incorporated selected quantitative and qualitative questions from the Medical Expenditure Panel Survey (MEPS)—Access to Care Section and selected questions from a previous Texas study that examined mixed-status households' health care seeking experiences in Lower Rio Grande Valley of South Texas (18). The questionnaire was administered in Spanish by the lead researcher. The interview questionnaire was pilot tested on participants prior to being used.

### Recruitment

Purposeful sampling was appropriate for this study. Identification and recruitment of eligible mixed-status household members was done via outreach to immigrant-serving organizations in the Tucson area as well as local health clinics, promotoras (community health workers), the Mexican Consulate's Ventanilla de Salud, public libraries, and word of mouth. Flyers in both English and Spanish were posted in key locations were families would often frequent such as public libraries, local school bulletins, family resource centers and shopping centers etc. To be eligible to participate in the study, individuals had to be over 18 years of age, reside in Tucson, be a primary decision maker of the family's healthcare needs and belong to a Latino household with at least one household member of a different immigration status. For purposes of this study *non-Latino* immigrant households were excluded. Interested members were screened for eligibility criteria over the phone or in person when possible. Eligible individuals were then scheduled for an interview at a convenient time and location of their choosing. Most interviews took place at local neighborhood center or convenient public library locations. Verbal consent was obtained before semi-structured interviews were conducted. Interviews varied between 15 and 60 min in length. Interviewees were compensated $25 in cash at the end of the interview session. Interviews were conducted in Spanish, audio recorded and transcribed for analysis.

### Analysis

Demographic data was captured via REDCap, a secure web application for building and managing online surveys. Transcriptions were completed in Spanish and data analysis was conducted using qualitative software Nvivo 10. Deductive and inductive content analysis was used to analyze data. Variables from a previous feasibility study were used to conduct the deductive analysis and inductive analysis was conducted with emerging *new* themes (32).

## Results

Forty-three adults from mixed-status households were interviewed. The majority of the interviewees came from a household where at least one household member was undocumented (81%). As illustrated by Table 1, 84 percent of interviewees identified as female while only 16% identified as male; 51% of interviewees were between the ages of 35–49; 98% were foreign born with 67% identifying as undocumented. Forty-two percent reported obtaining their usual source of care at a federally qualified health center under the discount care program while 21% of interviewees reported not having a usual place for care (Table 1). The majority of interviewees described their health as either good (42%) or fair (30%). Thirty percent of interviewed households consisted of households with a child under the age of 5 while 67% reported having minors under 18 years old as part of their household. However, a significant concern is raised in terms of the proportion of the sample that reported their health as “fair” (30%) or “poor” (12%). This combined percent of 42% also reported “no usual source of health care,” 23 and 40%, respectively.

**Table 1:** Interviewee's usual source of health care^a^ and selected demographic (*N* = 43).

**Demographic Characteristics**	***N***	**%**	**Medicaid (AHCCCS)**		**Employer- based coverage**		**PCAP**		**FQHC—Discount care program**		**Free clinics**		**No usual source of health care**	
Total	43	100	5	12%	4	9%	3	7%	18	42%	4	9%	9	21%
**AGE**														
18–24 years	2	5	1	50%					1	50%				
25–34 years	6	14	1	17%	2	33%	1	17%	2	33%				
35–49 years	22	51	1	5%			1	5%	13	59%	4	18%	3	14%
50 years and over	13	30	2	15%	2	15%	1	8%	2	15%			6	46%
**SEX**														
Female	36	84	3	8%	4	11%	2	6%	16	44%	4	11%	7	19%
Male	7	16	2	29%			1	14%	2	29%			2	29%
**IMMIGRATION STATUS**														
Foreign Born	42	98	4	10%	4	10%	3	7%	18	43%	4	10%	9	21%
U.S. Born	1	2	1	100%									
U.S. Citizen	7	16	5	71%	1	14%							1	14%
LPR	5	12			2	40%			2	40%			1	20%
Special Visa	1	2											1	100%
DACA	1	2			1	100%								
Undocumented	29	67					3	10%	16	55%	4	14%	6	21%
**MARITAL STATUS**														
Married	30	70	3	10%	3	10%	3	10%	16	53%	3	10%	2	7%
Separated	1	2							1	100%			
Divorced	3	7											3	100%
Widowed	1	2											1	100%
Single	7	16	2	29%					1	14%	1	14%	3	43%
Co-habitating	1	2			1	100%								
**PERCEIVED HEALTH**														
Excellent	2	5							1	50%	1	50%		
Very Good	5	12							3	60%			2	40%
Good	18	42	4	22%	1	6%	2	11%	8	44%	1	6%	2	11%
Fair	13	30			3	23%	1	8%	5	38%	1	8%	3	23%
Poor	5	12	1	20%					1	20%	1	20%	2	40%
With at least 1 undocumented h member^**^	35	81												
With at least 1 h member under <5 years	13	30												
With at least 1 minor h member between 5 and 17 years	29	67												

a *Health care accessibility (or access to care) is defined in this study as the presence of a location or person that family members go for routine preventative care, urgent care, medical treatment when sick and/or to seek health advice/consult*.

** *Includes self-reported undocumented status*.

### Barriers to Care

Fifty-three percent of interviewees reported having difficulty in obtaining health coverage with 57% of interviewees listing complexity of application requirements (paperwork) as the main reason for having difficulty in obtaining coverage; 26% listed discrimination and fear while 13% reported wait times as factors. Other reasons reported related to cost of care, confusing health plans among other logistical barriers in obtaining care (Table 2).

**Table 2:** Difficulty obtaining health coverage^a^ and selected demographic characteristics (*n* = 23).

	**Experienced difficulty**		**Reason(s) for Difficulty**							
**Demographic characteristics**	***n***	**%**	**Complexity of application requirements**		**Discrimination fear**		**Wait times**		**Other**	
Total	23	53	13	57%	6	26%	3	13%	6	26%
**PERCEIVED DIFFICULTY (*****N*** **= 43)**										
Very difficult	9	21	6		2				2	
Somewhat difficult	12	28	5		3		1		4	
Not too difficult	2	5	2		1		2			
Not at all difficult	11	26								
No response	9	21								
**IMMIGRATION STATUS**										
U.S. Citizen	5	22	3		2		1		1	
Legal Permanent Resident	3	13	1						2	
Special Visa: VAWA	1	4							1	
Undocumented	14	61	9		4		2		2	
Foreign Born	22	96	12		6		3		6	
U.S. Born	1	4	1							
**USUAL SOURCE OF CARE**										
Medicaid (AHCCCS)	4	17	3		2		1			
Employer-based Coverage	2	9							2	
PCAP	3	13	3							
FQHC – Discount Care	9	39	6		2		1		1	
Free Clinics	2	9	1		1		1			
No usual source of care	3	13			1				3	

a *Health care accessibility (or access to care) is defined in this study as the presence of a location or person that family members go for routine preventative care, urgent care, medical treatment when sick and/or to seek health advice/consult*.

#### Complexity of Application Requirements—Verification Criteria and Eligibility

Households reported the application process as a main barrier to care. They described the amount of paperwork required to apply for coverage as complex and insurmountable and felt that the application criteria did not take their specific circumstances into account. Paperwork, specifically for those applying to the Arizona Health Care Cost Containment System (AHCCCS), consisted of back and forth requests from the Department of Economic Security (DES). Furthermore, if eligible, the wait time to attain coverage took several weeks up to months. Interviewees explained that due to the nature of their employment, unsteady and informal in nature, they would often lack proper income documentation required as part of the application process as described below:

*I recall at that time, they asked so many questions. And since we don't have legal status that is what is problematic, because they ask for paystubs to prove income to see if you qualify for discount care and all that. And that's where it's challenging because then I tell them, ‘My husband is self-employed, he isn't paid with checks.’ Then we had to take [verification] letters and they had to be notarized and to notarize them they ask you for a State ID*.

Another issue related to how eligibility is determined based on household size and income ratio, these criteria do not necessarily take into account the complex nature of mixed-status households as it fails to consider existing family members that are physically separated due to deportation or detention. Therefore, the household size reported is not an accurate depiction of the *household* information criteria when households are still financially supporting family members that are living across the border (e.g., in Mexico) or in detention centers.

“*Due to the separation [husband's]. In fact, that bill, of $1,500, is still pending because I was never able to pay it. But they didn't provide my son AHCCCS because supposedly I earned too much, but the money goes to support them and my husband in Mexico.”*

#### Fear and Discrimination

Content analysis indicated that fear and discrimination continue to be a recurring factor 5 years after SB1070 was enacted. Fears were primarily related to concerns of deportation and/or detention leading to separation of family members when seeking public services. The fear is so great that in certain cases, care was delayed. In other, more extreme cases, undocumented family members avoided care completely even when experiencing critical health problems (e.g., facial paralysis, shortness of breath, and numbing of limbs). Jazmin, a 49-years old legal permanent resident and mother of three—a U.S. born daughter, a son with Deferred Action for Childhood Status (DACA), and an undocumented son and husband, described her dilemma in seeking medical services for her undocumented husband and son who were experiencing numbness and tingling of feet, chest pain, shortness of breath the day prior yet refused medical treatment out of fear. She discusses how it emotionally impacts her and the family as a whole.

“*In the current case… like yesterday, I was hysterical because I said, ‘What am I to do in case of an emergency [medical]?’ [Breaks down crying] I can't just take off to a hospital…yesterday's midday ordeal was very difficult. That I said [to self] if he's [husband] working I will go pick him up, then what I am I to do at home if he has a stroke? I will have to return to Mexico with him. He of course doesn't want to go back, ‘What for? There's nothing in Mexico.’*

*Yesterday was a difficult experience and last night was another experience…I got scared…[Eldest son] has over 20 years that he has not gone for a check-up, no vaccines, not even for TB nor Hepatitis, nothing. We are on edge all the time that we don't have anything [health wise]. ‘Let's go get your check-up at health fairs?’ I ask [Eldest son]*,

‘*No, I don't have anything! I know they'll ask me for names and I don't want that public.' So they [son and husband] are clinging to that idea, like in hiding. I tell them ‘We can't continue to be like this.’ We are living in hysteria.”*

Jazmin's story illustrates the challenges that households' face, particularly mixed-status households, who are not only divided by their immigration status but also in their access to health care services. Despite the fact that Jazmin recently obtained legal permanent residence she further explains what being part of a mixed-status family is like,

“*So, this is why I say, it's somewhat difficult [having the two undocumented family members]. Despite the fact that we have legal documentation, it's hard for me and my other children as well…I feel a lack of control despite the fact that I just got my immigration papers, at times I feel terrified, like yesterday I asked ‘What do I do?’”*

Another 37-years old undocumented mother of three children—two U.S. born children and an older daughter born in Mexico explains her predicament when tending to her children's care.

“*It takes a while to take my eldest daughter to the dentist. Sometimes I don't have the funds to pay, because even though I have PCAP, I have to pay $25. That in addition to dental services, that at times are up to $35-$60 dollars…Sometimes it can be up to two years that I don't get her cleaning. She tells me, ‘Why do you take my brother and not me?…It's been like that since she was younger, since she was about six she would say, ‘Why do you take my brother to the dentist all the time and not me?’…She would get upset with her brother.”*

Perceived discrimination was reported as a barrier when seeking health care services for eligible household members. In some instances interviewees reported being asked for documentation despite the fact that they were seeking services for other eligible family members and not themselves.

#### Wait Times

Wait times was another contributing factor reported by households in accessing healthcare. Long wait times had to do with the processing of applications to obtain coverage while in other cases wait times included primary care, specialty and urgent care appointments. In some cases households had difficulty submitting verification requirements or did not have funds to pay for discount membership fees such as PCAP, which ultimately delayed their approval hence care.

“*In the last year my husband obtained it [discount program] but we had to wait, to save money. During that time he didn't have health insurance. His primary doctor ordered an exam because he was experiencing vertigo, he ordered a CAT Scan. He didn't have any coverage so I had to help him apply for PCAP [discount program] and then he applied but it's a process and they don't give it to you immediately. We waited about 2 months to do the CAT Scan.”*

#### Health Literacy

Other barriers conveyed by households relate to cost of care, confusion over eligibility of care and misunderstanding regarding coverage such as limitations of discount programs and/or emergency AHCCCS coverage.

### Promoters to Care

When asked what they found helpful in obtaining coverage 70% of interviewees reported multiple reasons that assisted them in obtaining coverage (Table 3); 43% reported affordability of care; 37% reported responsive and accommodating front-line staff; 20% reported co-location of services and 17% listed assistance with applications. Other findings related to proximity of location, language availability, ease of appointments and employer based assistance with insurance. Of the 13 interviewees that reported affordability of care, 10 obtained coverage via FQHC's discount program. Recipients of the FQHC's discount program also reported co-location of services as a factor for obtaining coverage.

**Table 3:** Promoters to obtaining health coverage^a^ and selected demographic characteristics (*n* = 30).

	**Promoters to Obtaining Care**		**Reason(s) for ease in obtaining care**									
**Demographic characteristics**	***n***	**%**	**Affordability of care**		**Front-line Staff**		**Co-location of services**		**Assistance with application**		**Other**	
Total	30	70	13	43%	11	37%	6	20%	5	17%	13	33%
**IMMIGRATION STATUS**												
U.S. Citizen	4		2				1		3			
Legal Permanent Resident	3	10	3		1				1		2	
DACA	1	3									1	
Special Visa: VAWA	1	3	1						1			
Undocumented	21	70	9		8		6		2		7	
Foreign Born	29	97	13		11		6		4		13	
U.S. Born	1	3							1			
**USUAL SOURCE OF CARE**												
Medicaid (AHCCCS)	4	13			2				1		3	
Employer-based Coverage	2	7									2	
PCAP	3	10			2						2	
FQHC—Discount Care	15	50	10		5		6		3		4	
Free Clinics	4	13	2		1						2	
No usual source of care	2	7	1		1				1			

a *Health care accessibility (or access to care) is defined in this study as the presence of a location or person that family members go for routine preventative care, urgent care, medical treatment when sick and/or to seek health advice/consult*.

#### Affordability of Care

Safety net programs continue to be critical spaces to provide healthcare services for low-income community members including immigrant households. Discount programs at community health clinics such as discount-care programs offered by Federally Qualified Health Centers (FQHC) or the Pima County Access Program (PCAP) provide many households the possibility of obtaining care at reduced costs albeit with limited care options (i.e., specialty care). While high cost of care remains a factor for many households, they explained that discount programs helped them access care at much more affordable costs.

“*So it is there [FQHC] that it's been more economical because they only charge us $25 per visit cost plus the medicine. And the medication is also discounted making it very economical. It's really great. I can't complain, we don't complain about that. When they no longer are able to assist us that's when it's difficult. When it's no longer in their hands, then yes [it's problematic].”*

#### Front Line Staff

Interviewees reported that experiences with front-line staff and health providers made a huge difference in attainment of services. Some households shared that after a bad experience with front line staff, friends informed them about a different location where staff were much friendlier and reportedly did not discriminate against immigrant households. In such cases, they would present the same documentation but staff worked with them offering different options to provide needed verification. In essence front line staff were more responsive and accommodating to households making it possible for them to obtain services.

#### Co-location of Services

Households mentioned that obtaining care was facilitated when all members of the household, regardless of immigration status, could seek care at the same clinic site. Additionally the attainment of co-located services such as primary care, OB/GYN, pediatric care, provision of medications and lab tests were at the same location made a huge difference in their care. As Martha, a 35-years old undocumented mother of two explains that she and her husband, who is also undocumented, plus the children are all seen at the same clinic.

“*Well I go because we're all there, I'm there and so are my children.”*

#### Assistance With Applications

Another important factor reported in attaining coverage was the assistance in completing application forms. This was particularly helpful when applying for AHCCCS. Households sought assistance with applications at FQHC, community centers such as the Food Bank, and at the Ventanilla de Salud. They mentioned that when they applied and obtained assistance enrollment was much smoother. An example is of an income affidavit where households do not have to present pay stubs but have a notarized document making it possible for them to apply for services.

“*She took about 3 hours entering all the information [in the system] then she told me, ‘You know? It won't allow me yet, but I will call you tomorrow to see if you qualified.' And yes, the next day, very amicably, she called me to tell me, ‘You know, you qualified and it's all done,' … but I tell you there's a big difference between one clinic and the other, or one service and another*.

## Discussion

Study findings indicate that fear, distrust and trepidation in seeking care continues to be of concern to immigrant households in Tucson, Arizona 5 years post SB1070. More notably, the effects of prolonged stress, anxiety and trauma remain unaddressed whilst mental health services continue to be absent in the care they receive. Additionally mixed-status households continue to face special challenges in accessing care (16, 33, 34). This holds particularly true when family members are separated due to deportation and/or detention although the household continues to financially support them. The complexities of application requirements to verify eligibility make it even more challenging due to their predicament. Households reportedly made several attempts to provide needed paperwork delaying attainment of health coverage. The bureaucratic barriers, such as the front-line staff's discretionary application of rules and procedures, at times impeded while other times promoted the attainment of services. Hacker et al. support similar findings in a recent literature review on barriers to care to undocumented immigrants (35).

Barriers reported by households continue to relate to discrimination and fear, complexity of application requirements (paperwork) as the main reasons for having difficulty in obtaining coverage while others reported wait times as a factor. Reports of fear, stress and anxiety experienced by households have surfaced in a previous study (36), conditions that remain unaddressed, as mental health services are virtually non-existent. Results also indicate that local programming is a vital source of care for mixed-status households. Safety net programs and discount care programs provide affordability of care. The discretionary practices exercised by front-line staff to assist with applications is another, albeit informal, promoter of health care access. Interview results provide an in-depth understanding of mixed-status households' accessibility to care in the southwest region. Additional research is needed to examine how households will continue to respond to increased marginalization from health care programs. We advocate for local response to sensible positions on immigration policy relating to health care accessibility that include mental health services.

Despite reported barriers, only 21% households reported not having a source of care and 79% reported having an existing place for care. The type of coverage mixed-status households reported using was primarily public coverage over private insurance. In many instances households were willing to pay into discount care programs and/or safety net programs to obtain needed care. Although the uses of safety-net programs are available, they have limitations in terms of care particularly in providing specialty care. Immigrant households therefore could benefit greatly in the inclusion of health care insurance coverage.

This study also identified several factors that promote care among mixed-status households. These include the provision of discounted services via safety net programs and personalized discretionary attention exercised by front-line workers in providing assistance in navigating the complex application process and forms needed for enrollment in health care programs. Findings have implications beyond Arizona specifically in the last few years as anti-immigrant policies and rhetoric have extended beyond the State, influencing the national debate. During and after the presidential campaign, anti-immigrant rhetoric and ICE raids have reignited fear and distrust among many immigrants throughout the country (37). Recently, the Homeland Security Department under the Trump Administration announced a proposed rule that would make changes to “public charge” policies. Public charge policies govern how the use of public benefits can affect an individuals' ability for lawful admission to the U.S. or adjust to legal permanent resident status. If a public charge determination is made, the government may deny non-citizens lawful admission or lawful permanent resident status. Under current policy, public charge only covers non-citizens who are primarily reliant on cash benefit assistance. Under the proposed rule, officials would now consider use of certain previously excluded programs in public charge determinations which include healthcare assistance programs such as Medicaid, the Medicare Part D Low-Income Subsidy Program, the Supplemental Nutrition Assistance Program in addition to housing programs (38). It is anticipated that these changes may lead to broad declines in participation in social programs among eligible immigrant households and their primarily U.S.-born children beyond those directly affected by the changes (39).

We argue for strong local response to restrictive legislation toward the achievement of health equity in immigrant communities. In efforts to continue to address and expand access to care to mixed-status households, recommendations include the expansion of safety net programs and training of healthcare professionals and front-line staff to address the unique needs of mixed-status households in the provision of care. Additionally there is a need for increased outreach to immigrant households to provide health literacy programming and know your health rights workshops to facilitate usage and assist in the navigation of healthcare programs to gain a better understanding of health systems. Ultimately continued advocacy for immigration reform and inclusivity in healthcare is at the heart of achieving health equity.

### Study Limitations

Generalizability was not the intent of this study. Although this study provides an insight to mixed-status households' health care behaviors, it is with limitations. Random sampling was not possible because the number of mixed-status households is unknown. Moreover, access to this population is often difficult due to their concerns of being identified and deported.

## Ethics Statement

This study was carried out in accordance with the recommendations of the University of Arizona's Institutional Review Board with informed consent from all subjects. All subjects provided informed consent in accordance with the Declaration of Helsinki. The protocol (#1509115072) was approved by the University of Arizona's Institutional Review Board.

## Author Contributions

SG is primary author and AOO is secondary author. We agree to be accountable for the content of the work.

### Conflict of Interest Statement

The authors declare that the research was conducted in the absence of any commercial or financial relationships that could be construed as a potential conflict of interest.
